# Multienzyme interactions of the *de novo* purine biosynthetic protein PAICS facilitate purinosome formation and metabolic channeling

**DOI:** 10.1016/j.jbc.2022.101853

**Published:** 2022-03-21

**Authors:** Jingxuan He, Ling-Nan Zou, Vidhi Pareek, Stephen J. Benkovic

**Affiliations:** 1 Department of Chemistry, The Pennsylvania State University, University Park, Pennsylvania, USA; 2 Huck Institutes of the Life Sciences, The Pennsylvania State University, University Park, Pennsylvania, USA

**Keywords:** purinosome, *protein complex*, *de novo* purine biosynthesis, bimolecular fluorescence, complementation, metabolic channeling, co-immunoprecipitation, ADSL, adenylosuccinate lyase, ATIC, 5-aminoimidazole-4-carboxamide ribonucleotide formyltransferase/IMP cyclohydrolase, AICAR, 5-aminoimidazole-4-carboxamide ribonucleotide, BiFC, bimolecular fluorescence complementation, co-IP, co-immunoprecipitation, DNPB, *de novo* purine biosynthesis, FAICAR, 5-formamidoimidazole-4-carboxamide ribotide, GART, phosphoribosylglycinamide synthetase/formyltransferase/phosphoribosylaminoimidazole synthetase, IMP, inosine monophosphate, PPI, protein–protein interaction, PAICS, phosphoribosylaminoimidazole carboxylase/succinocarboxamide synthetase, crPAICS, PAICS-knockout HeLa cells, PPAT, amidophosphoribosyltransferase, PFAS, phosphoribosylformylglycinamidine synthase, SAICAR, phosphoribosylaminoimidazolesuccinocarboxamide, TBS, tris-buffered saline, THF, tetrahydrofolate

## Abstract

There is growing evidence that mammalian cells deploy a mitochondria-associated metabolon called the purinosome to perform channeled *de novo* purine biosynthesis (DNPB). However, the molecular mechanisms of this substrate-channeling pathway are not well defined. Here, we present molecular evidence of protein–protein interactions (PPIs) between the human bifunctional phosphoribosylaminoimidazole carboxylase/succinocarboxamide synthetase (PAICS) and other known DNPB enzymes. We employed two orthogonal approaches: bimolecular fluorescence complementation, to probe PPIs inside live, intact cells, and co-immunoprecipitation using StrepTag-labeled PAICS that was reintegrated into the genome of PAICS-knockout HeLa cells (crPAICS). With the exception of amidophosphoribosyltransferase, the first enzyme of the DNPB pathway, we discovered PAICS interacts with all other known DNPB enzymes and with MTHFD1, an enzyme which supplies the 10-formyltetrahydrofolate cofactor essential for DNPB. We show these interactions are present in cells grown in both purine-depleted and purine-rich conditions, suggesting at least a partial assembly of these enzymes may be present regardless of the activity of the DNPB pathway. We also demonstrate that tagging of PAICS on its C terminus disrupts these interactions and that this disruption is correlated with disturbed DNPB activity. Finally, we show that crPAICS cells with reintegrated N-terminally tagged PAICS regained effective DNPB with metabolic signatures of channeled synthesis, whereas crPAICS cells that reintegrated C-terminally tagged PAICS exhibit reduced DNPB intermediate pools and a perturbed partitioning of inosine monophosphate into AMP and GMP. Our results provide molecular evidence in support of purinosomes and suggest perturbing PPIs between DNPB enzymes negatively impact metabolite flux through this important pathway.

Most metabolic enzymes are part of large metabolic networks where multiple enzymes, of varying substrate affinities and enzymatic activities, must work in concert. Thus, the biochemistry of isolated enzymes, studied *in vitro*, provides only partial pictures of their behavior in their native context inside living cells. Indeed, *in vivo* studies have increasingly demonstrated the importance of protein–protein interactions (PPIs) between enzymes, and between enzymes and other proteins and cellular structures, in regulating cell metabolism (1, 2, 3, 4, 5, 6, 7, 8). These interactions can enhance flux by channeling intermediate products between successive enzymes, limit the loss of unstable and/or the accumulation of toxic intermediates, and alter enzymatic activities and/or ligand-binding affinities (9). The identification of new multienzyme metabolic complexes, or “metabolons”, revealing their composition and modes of regulation, promises to re-shape our understanding of basic cellular metabolic processes.

*De novo* purine biosynthesis (DNPB) is an ancient and highly conserved pathway, whose products are essential for nearly all aspects of cell biology (10, 11). In humans, DNPB generates inosine monophosphate (IMP) from small molecule precursors in 10 reactions catalyzed by six enzymes: amidophosphoribosyltransferase (PPAT), phosphoribosylglycinamide synthetase/formyltransferase/phosphoribosylaminoimidazole synthetase (GART), phosphoribosylformylglycinamidine synthase (PFAS), phosphoribosylaminoimidazole carboxylase/succinocarboxamide synthetase (PAICS), adenylosuccinate lyase (ADSL), and 5-aminoimidazole-4-carboxamide ribonucleotide formyltransferase/IMP cyclohydrolase (ATIC) (Fig. 1) (12). Motivated by multiple lines of evidence, this pathway is hypothesized to require a multienzyme complex—the purinosome—for optimal flux. 5-phosphoribosylamine, the product of the first reaction in DNPB, is highly unstable (13, 14); the direct transfer of 5-phosphoribosylamine from PPAT to GART should minimize its loss. Reactions 2, 3, 5 are catalyzed by the trifunctional enzyme GART, but reaction 4 is catalyzed nonsequentially by PFAS; this suggests GART and PFAS interact to facilitate the back-and-forth of intermediates. 5-aminoimidazole-4-carboxamide ribonucleotide (AICAR), the product of reaction 8, is an activator of AMPK as well as an inhibitor of mTOR and can negatively impact cell proliferation (15, 16, 17, 18, 19); since the enzyme ADSL catalyzes the interconversion of phosphoribosylaminoimidazolesuccinocarboxamide (SAICAR) and AICAR in a reversible manner, the interaction of ADSL with the respective upstream/downstream enzymes in the pathway may be important in regulating SAICAR/AICAR accumulation. In reaction 9, ATIC converts AICAR to 5-formamidoimidazole-4-carboxamide ribotide (FAICAR), while *in vitro* studies indicate ATIC favors the back-conversion of FAICAR to AICAR (20, 21); the rapid conversion of FAICAR into IMP by a DNPB metabolon may be critical for driving the forward AICAR to FAICAR conversion.

**Figure 1:**
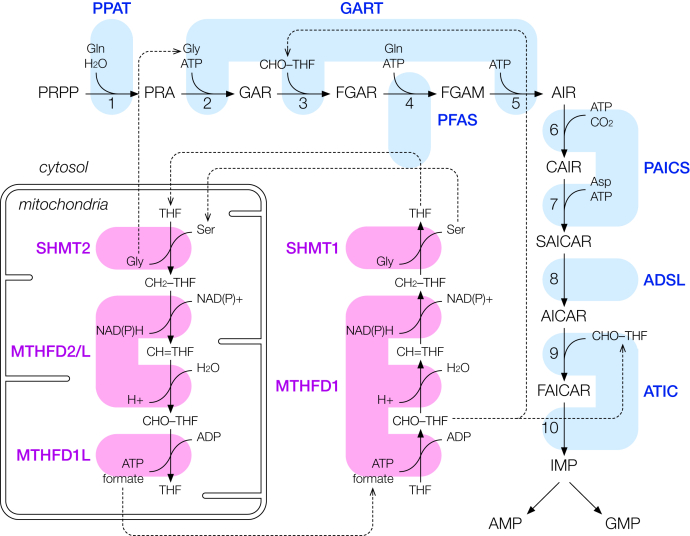
**Schematic of the human *de novo* purine biosynthesis (DNPB) pathway.** The six DNPB enzymes (*cyan*) convert phosphoribosyl pyrophosphate (PRPP) to inosine monophosphate (IMP) in a sequence of 10 reactions: amidophosphoribosyltransferase (PPAT) converts PRPP to 5-phosphoribosylamine (PRA); PRA is converted by trifunctional phosphoribosylglycinamide synthetase/formyltransferase/phosphoribosylaminoimidazole synthetase (GART) to glycinamide ribonucleotide (GAR), and then to phosphoribosyl-N-formylglycineamide (FGAR); phosphoribosylformylglycinamidine synthase (PFAS) converts FGAR to 5-phosphoribosylformylglycinamidine (FGAM), which is then converted by GART to 5-aminoimidazole ribotide (AIR); AIR is converted by bifunctional phosphoribosylaminoimidazole carboxylase/succinocarboxamide synthetase (PAICS) to 5-phosphoribosyl-4-carboxy-5-aminoimidazole (CAIR), and then to phosphoribosylaminoimidazolesuccinocarboxamide (SAICAR); SAICAR is converted to 5-aminoimidazole-4-carboxamide ribonucleotide (AICAR) by adenylosuccinate lyase (ADSL); finally, AICAR is converted to 5-formamidoimidazole-4-carboxamide ribotide (FAICAR) and then IMP by the bifunctional 5-aminoimidazole-4-carboxamide ribonucleotide formyltransferase/IMP cyclohydrolase (ATIC). Also shown (*magenta*) are the mitochondrial (serine hydroxymethyltransferase [SHMT2], bifunctional methylenetetrahydrofolate dehydrogenase/cyclohydrolase [MTHFD2/2L], formyltetrahydrofolate synthetase [MTHFD1L]) and cytosolic (serine hydroxymethyltransferase ([SHMT1], trifunctional methylenetetrahydrofolate dehydrogenase/cyclohydrolase/formyltetrahydrofolate synthetase [MTHFD1]) one-carbon metabolism enzymes responsible for the interconversion between tetrahydrofolate (THF), 5,10-methylenetetrahydrofolate (CH_2_-THF), 5,10-methenyltetrahydrofolate (CH=THF), and 10-formyltetrahydrofolate (CHO-THF)—the last being a necessary cofactor for DNPB.

Imaging studies provided early support for the purinosome hypothesis. DNPB enzymes, tagged with fluorescent proteins, transiently co-expressed in purine-starved HeLa cells, were found to colocalize in submicron punctate bodies (22). These bodies also co-localize with microtubules and mitochondria, the latter possibly reflecting DNPB's dependence on mitochondrially derived formate (23, 24, 25). More recently, metabolic profiling of the DNPB pathway, using isotopically labeled serine as tracer, found the distribution of labeled products that is inconsistent with the presence fully equilibrated, freely diffusing intermediates, thereby providing compelling evidence for intermediate channeling (26). In those experiments, serine is processed by mitochondrial one-carbon metabolism enzymes to produce DNPB substrates glycine and formate. Metabolic profiling indicated the end product purine nucleotides (AMP and GMP), synthesized by purinosome-mediated channeled DNPB, preferentially incorporated mitochondrially generated substrates, suggesting purinosomes directly take up mitochondrial formate before it can mix and equilibrate with the cytosolic formate pool. Since the cytosolic NAD(P)H/NAD(P)+ ratio favors the formation of the reduced form of the tetrahydrofolate (THF) cofactor (methylene-THF, CH_2_-THF) rather than the oxidized form (formyl-THF, CHO-THF) required for DNPB (Fig. 1) (27, 28), the cytosolic formyltetrahydrofolate synthetase MTHFD1 is proposed to be part of the purinosome to enable the direct uptake of mitochondrial formate and to ensure the effective incorporation of CHO-THF into DNPB before it is reduced. While biochemical fractionation experiments have found that several DNPB enzymes can be co-purified, suggesting they may interact (29, 30, 31), direct physical evidence for PPIs between DNPB enzymes has been limited, and interactions between MTHFD1 and DNPB enzyme have not been previously reported. Thus, a definitive identification of the purinosomes complex and its constituents remains elusive.

Additionally, one can ask whether the assembly of the purinosome proceeds through the condensation of preexisting binary or higher-order complexes. Are complete purinosomes formed only when the cells are cultured in purine-depleted conditions or are they (or their subcomplexes) always present regardless of the state of the DNPB pathway? Here, we probe the PPIs of the bifunctional enzyme PAICS, which catalyzes reactions 6 and 7 of the DNPB pathway. Notably, PAICS is overexpressed in multiple cancers (32, 33, 34, 35, 36, 37), suggesting it may have a pivotal role in increasing the flux of the DNPB pathway to meet the elevated purine demand of rapidly proliferating cancer cells. We deploy two orthogonal approaches: bimolecular fluorescence complementation (BiFC), to probe PPIs in intact living cells, and co-immunoprecipitation (co-IP), the “gold-standard” molecular probe for PPIs. We find that PAICS interacts with all other DNPB enzymes with the exception of PPAT, the first enzyme in the pathway. PAICS also interacts with MTHFD1, whose activity yields essential CHO-THF cofactor for DNPB. Furthermore, we observed these interactions in cells cultured under both purine-rich and purine-depleted conditions, indicating the purinosome, at least as a partial complex, is present regardless of the DNPB state of the cell. We also found that the tagging of PAICS on its C terminus resulted in an enzyme whose catalytic activities are intact, but whose PPIs are markedly deficient. Interestingly, crPAICS cells rescued by the integration of C-terminal–labeled PAICS-2×Strep (denoted crPAICS::PAICS-2×Strep) grow slower than crPAICS rescued with N-terminal tagged 2×Strep-PAICS (denoted crPAICS::2×Strep-PAICS), despite a higher level of PAICS protein expression. Consistent with this observation, metabolic profiling indicates while crPAICS::2×Strep-PAICS exhibits signatures of channeled DNPB similar to that of WT HeLa, crPAICS::PAICS-2×Strep exhibits reduced DNPB flux and an aberrant partition of IMP between guanine and adenine nucleotides. Together, our results demonstrate the presence of PPIs between DNPB enzymes—a prerequisite for the assembly of the purinosome and provide evidence that the disruption of these PPIs has a profoundly negative impact on the output of this critical pathway.

## Results

### BiFC reveals PAICS interactions with other DNPB enzymes

Imaging studies showed the PFAS puncta observed in purine-starved HeLa cells co-localize with mitochondria and with the microtubule cytoskeleton. We are concerned by the possibility that PPIs between DNPB enzymes may depend on these structures as scaffolds and would not survive cell lysis. We therefore chose BiFC to probe potential PPIs between DNPB enzymes in live, intact cells.

**Figure 2:**
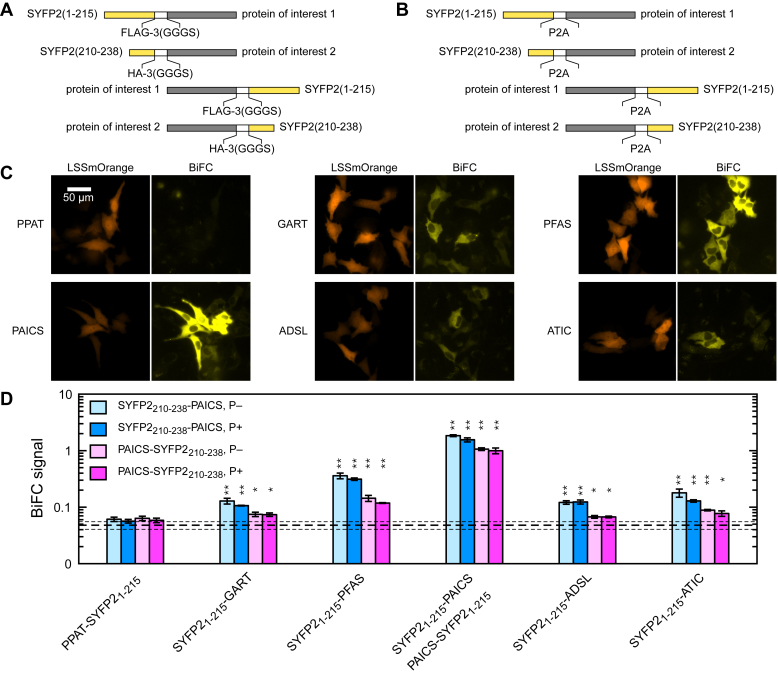
**Probing PPIs between PAICS and other DNPB enzymes using BiFC.** Design schematic for plasmid expression cassettes encoding (*A*) BiFC assay and (*B*) BiFC control fusion constructs. For the BiFC control constructs, the linker between the SYFP2 fragment and the DNPB enzyme is replaced with a self-cleaving P2A peptide. *C*, representative images showing the reconstituted SYFP2 signal in HeLa cells cultured due to the interaction of SYFP2_210-238_-PAICS with PPAT-SYFP2_1-215_, SYFP2_1-215_-GART, SYFP2_1-215_-PFAS, SYFP2_1-215_-PAICS, SYFP2_1-215_-ADSL, and SYFP2_1-215_-ATIC. Here, LSSmOrange labels cells that have been successfully transfected with the BiFC expression vectors. *D*, quantitated BiFC signal in HeLa cells between PAICS with all the DNPB enzymes. *Dashed lines* indicate background BiFC signal ± standard deviation, as obtained from self-cleaving P2A control constructs. *p*-values from unpaired *t* tests comparing BiFC signal *versus* P2A controls: ∗ <0.01, ∗∗ <0.001. ADSL, adenylosuccinate lyase; ATIC, 5-aminoimidazole-4-carboxamide ribonucleotide formyltransferase/IMP cyclohydrolase; BiFC, bimolecular fluorescence complementation; DNPB, *de novo* purine biosynthesis; GART, phosphoribosylglycinamide synthetase/formyltransferase/phosphoribosylaminoimidazole synthetase; PAICS, phosphoribosylaminoimidazole carboxylase/succinocarboxamide synthetase; PFAS, phosphoribosylformylglycinamidine synthase; PPI, protein–protein interaction; PPAT, amidophosphoribosyltransferase.

In BiFC, potential interactors are tagged with complementary, nonfluorescent, fragments of a fluorescent protein. Upon interaction, these complementary fragments are brought into proximity to reconstitute an intact fluorescent protein (38). We chose as BiFC tags two fragments of SYFP2 (39), a bright YFP variant: SYFP2_1-215_ and SYFP2_210-238_ (Fig. 2*A*). A similar fragmentation of mVenus (which differs from mVenus by a single residue V68L) had been identified as a sensitive BiFC pair in a systematic screen (40, 41, 42). Because BiFC becomes irreversible upon fluorophore maturation, fast-maturing SYFP2 (maturation time of 4.1 min *versus* 17.6 min for mVenus) should enable the detection of more transient PPIs (43). We constructed BiFC expression vectors by tagging all six DNPB enzymes with complementary SYFP2 fragments, connected by a linker containing a flexible repeat (3×GGGS) and an epitope tag (FLAG-tag or HA-tag). We also constructed control vectors, where the linker between the SYFP2 fragment and the DNPB enzyme is replaced with a self-cleaving P2A peptide (44) (Fig. 2*B*). Thus, for each BiFC pair, we can conduct a matched control to assess the background level of spontaneous SYFP2 reconstitution. Only BiFC signals that are significantly above this background can be considered as a positive indication of PPI.

To assess DNPB enzyme pairs for potential PPI, equal amounts of paired BiFC vectors are cotransfected with a vector expressing LSSmOrange (45), into WT HeLa. LSSmOrange, which is well separated from SYFP2 in both excitation and emission, serves as a transfection label. Twenty-four hour posttransfection, we image transfected cells and analyze the results with automated image analysis. We evaluate the BiFC signal as the mean SYFP2 fluorescence in cells with above-background levels of LSSmOrange fluorescence, indicating they have been successfully transfected (Fig. 2*C*). Using PAICS tagged on its N terminus with SYFP2_210-238_, we were able to detect robust BiFC signals with SYFP2_1-215_-tagged GART, PFAS, PAICS, ADSL, and ATIC. In all cases, the observed BiFC fluorescence is predominantly diffuse and cytoplasmic. Interestingly, the BiFC signal of PAICS with PFAS is considerably stronger than that of PAICS with enzymes immediately upstream (GART) and downstream (ADSL) of it in the DNPB pathway. Only PPAT, the first enzyme in the DNPB pathway, failed to yield a significant BiFC signal (Fig. 2*D*).

We repeated the BiFC assay in both purine-depleted HeLa, where DNPB should be active, and in HeLa grown in purine-rich media, where the cellular purine demand is largely met by the salvage pathway; in both cases, we obtained essentially identical BiFC signals. We also noticed that the tagging PAICS at its C terminus with SYFP2_210-238_ fragment resulted in noticeably weaker BiFC signals with GART, PFAS, ADSL, and ATIC, whereas the BiFC signal with PAICS itself (C-terminal–labeled with SYFP2_1-215_) remained robust. Since PAICS is known to form a stable homo-octamer, this result suggests the heteromeric interaction of PAICS with other DNPB enzymes may be negatively impacted when PAICS is labeled on its C terminus.

### Stable integration of 2×StrepTag-labeled PAICS reconstitutes a functional DNPB pathway in PAICS-knockout HeLa and reveals extensive PPIs

Our BiFC results indicate that PAICS appears to interact with multiple DNPB enzymes. Moreover, the ability of BiFC to detect these interactions suggest they are not particularly transient, since only PPIs whose lifetimes are comparable or larger than the maturation time of SYFP2 can be expected to result in BiFC. While previous efforts using co-IP had not yielded clear evidence for PPIs between DNBP enzymes, our BiFC results suggest a renewed effort, focusing on PAICS, may yield positive results. Due to the lack of suitable antibodies, earlier efforts at co-IP relied on pulling down transiently expressed epitope-tagged DNPB enzymes from transfected cells (46). This likely leads to poor co-IP efficiency, as the large excess of overexpressed enzymes means many will remain uncomplexed. We therefore sought to generate cell lines that stably and exclusively express StrepTag-labeled PAICS at or below the endogenous PAICS expression level found in WT HeLa.

Previously, Zikanova *et al.* (47), using CRISPR-Cas9, had generated a PAICS-knockout HeLa cell line denoted crPAICS (48). These cells require purine supplementation for survival; upon purine depletion, they undergo cell cycle arrest and cell death. We therefore tested the ability of N- and C-terminal StrepTag-labeled PAICS to rescue these cells in purine-depleted conditions. We found that PAICS tagged at either terminus can maintain crPAICS survival in purine-depleted media, indicating that PAICS tagged at either terminus retained its essential enzymatic functions. In both cases, following extended culture in purine-depleted media, colonies emerged of cells that no longer depended on purine supplementation for survival. This indicates the transfected PAICS expression vector has been stably integrated into the genome, reconstituting a functional DNPB pathway. Using this strategy, we generated two stably rescued polyclonal cell lines crPAICS::2×Strep-PAICS and crPAICS::PAICS-2×Strep. In our experiments, the reintegration of PAICS expression (introduced *via* chemical transfection reagent) into the crPAICS is unaided by any retroviral machinery; it is thus a very low probability event, and we expect that the vast majority, if not all, of the rescued clones reintegrated just a single copy of PAICS.

**Figure 3:**
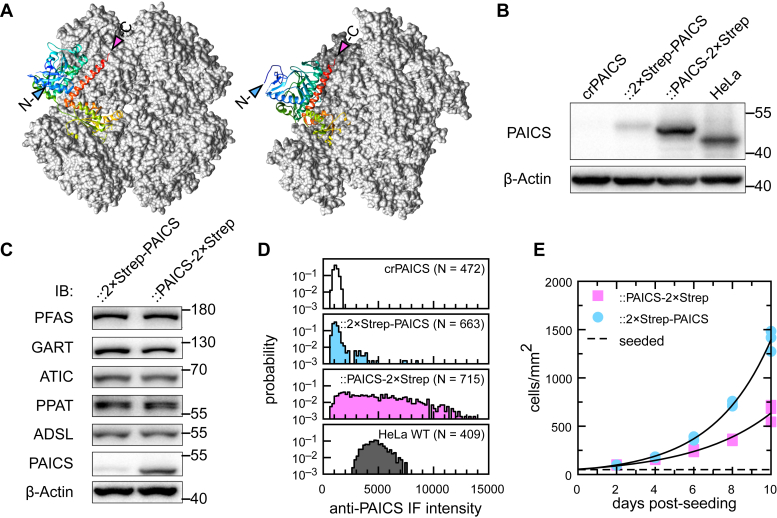
**Characterizing stably rescued PAICS-knockout HeLa (crPAICS) cells expressing StrepTag-labeled PAICS.***A*, frontal and oblique view of the crystal structure of human PAICS octamer (2H31). One PAICS monomer is rendered as a ribbon model, with locations of its N and C termini highlighted. *B*, Western blot analysis of reintegrated PAICS expression in rescued crPAICS cells. The expression of reintegrated PAICS in crPAICS::PAICS-2×Strep is ∼6 fold higher than that in crPAICS::2×Strep-PAICS. All cells except crPAICS were cultured in purine-depleted media. *C*, Western blot analysis comparing the expression of all six DNPB enzymes: PPAT, GART, PFAS, PAICS, ADSL, and ATIC between crPAICS::2×Strep-PAICS and crPAICS::PAICS-2×Strep cell lines. *D*, histograms of PAICS expression in single cells, measured from immunofluorescence images. *E*, cultured under purine-depleted conditions, stably rescued crPAICS::2×Strep-PAICS (doubling time 2.1 ± 0.1 days) proliferates at a faster rate than crPAICS::PAICS-2×Strep (doubling time 2.8 ± 0.2 days); *p*-value from unpaired *t* test <0.003. *Dashed line* indicates initial cell seeding density. *Solid lines* are fits to an exponential growth curve. ADSL, adenylosuccinate lyase; ATIC, 5-aminoimidazole-4-carboxamide ribonucleotide formyltransferase/IMP cyclohydrolase; DNPB, *de novo* purine biosynthesis; GART, phosphoribosylglycinamide synthetase/formyltransferase/phosphoribosylaminoimidazole synthetase; PAICS, phosphoribosylaminoimidazole carboxylase/succinocarboxamide synthetase; PFAS, phosphoribosylformylglycinamidine synthase; PPI, protein–protein interaction; PPAT, amidophosphoribosyltransferase.

Western blot analysis of these stably rescued cell lines found PAICS expression in crPAICS::2×Strep-PAICS is only one quarter that of WT HeLa (Fig. 3*B*), while that in crPAICS::PAICS-2×Strep is 1.4-fold higher; expression levels of the remaining DNPB enzymes are essentially identical across the two cell lines (Fig. 3*C*). Analysis of PAICS expression in individual cells using immunofluorescence (IF) found similar results. Both rescued cell lines showed much more heterogeneous cell-to-cell PAICS expression than WT HeLa (Figs. 3*D* and S1), as is expected from polyclonal cell lines. The large majority of crPAICS::2×Strep-PAICS cells express PAICS at very low levels, with IF signals comparable to the background IF signal from crPAICS; only a small fraction (∼4%) showed IF signals comparable to that in WT HeLa. On the other hand, ∼40% of crPAICS::PAICS-2×Strep cells exhibited PAICS expression higher (by up to 3×) than that in WT HeLa. In all cases (the two rescued cell lines and WT HeLa), anti-PAICS IF staining was diffuse, cytoplasmic, and showed no evidence of punctate structures even in the brightest-staining cells. Thus, even accounting for the heterogeneity in these polyclonal cell lines, crPAICS::2×Strep-PAICS express PAICS at significantly lower levels than crPAICS:PAICS-2×Strep. Surprisingly, crPAICS::2×Strep-PAICS, despite its lower PAICS expression, proliferated faster than crPAICS::PAICS-2×Strep (Fig. 3*E*). Our results show that PAICS expression at only a fraction of that found in WT HeLa is sufficient to sustain cell growth and proliferation in purine-depleted media. They also suggest that C-terminal–tagged PAICS-2×Strep, while capable of reconstituting a functional DNPB pathway, is nevertheless defective in some manner.

**Figure 4:**
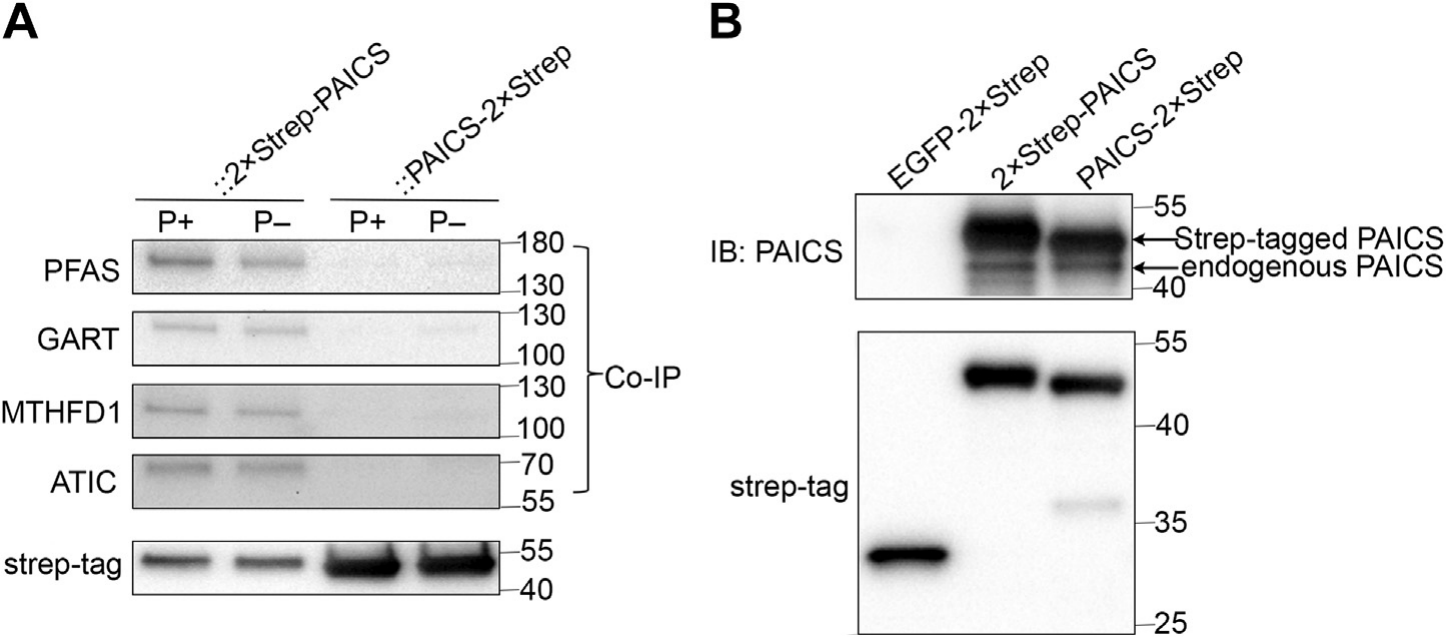
**Co-immunoprecipitation of DNPB enzymes with PAICS following anti-StrepTag pull-down from crPAICS::2×Strep-PAICS and crPAICS::PAICS-2×Strep cells.***A*, Western blots showing the co-IP of PFAS, GART, MTHFD1, and ATIC with StrepTag-labeled PAICS from rescued crPAICS cells cultured under both purine-rich (P+) and purine-depleted (P−) conditions. *B*, Western blots of endogenous PAICS co-immunoprecipitated by 2×Strep-PAICS or PAICS-2×Strep overexpressed in HEK293T; EGFP-2×Strep is used as the noninteracting co-IP control. ATIC, 5-aminoimidazole-4-carboxamide ribonucleotide formyltransferase/IMP cyclohydrolase; DNPB, *de novo* purine biosynthesis; GART, phosphoribosylglycinamide synthetase/formyltransferase/phosphoribosylaminoimidazole synthetase; PAICS, phosphoribosylaminoimidazole carboxylase/succinocarboxamide synthetase; PFAS, phosphoribosylformylglycinamidine synthase.

Next, we conducted anti-StrepTag affinity purification of cell lysates from these rescued cell lines, followed by Western blot analysis. For crPAICS::2×Strep-PAICS, we found ant-StrepTag pull-down co-precipitated GART, PFAS, and ATIC (Fig. 4*A*). We could not detect the co-precipitation of PPAT under any conditions, and we were unable to definitively identify the co-IP of ADSL because the anti-ADSL antibodies used cross-reacted with 2×Strep-PAICS and PAICS-2×Strep, whose Western band positions are close to that of ADSL. Furthermore, we found no difference in the co-IP of these enzymes in cells grown under purine-depleted *versus* purine-rich conditions. On the other hand, we could not reliably detect the co-precipitation of any DNPB enzyme for crPAICS::PAICS-2×Strep, despite a 6-fold higher PAICS expression. To examine the possibility that the apparently defective PPIs of C-terminal–tagged PAICS-2×Strep may be due to the destabilization of the PAICS homo-octamer, we overexpressed 2×Strep-PAICS and PAICS-2×Strep in HEK293T cells (grown in purine-depleted conditions) and examined their ability to co-IP endogenous PAICS. We found 2×Strep-PAICS and PAICS-2×Strep co-precipitated similar amounts of endogenous PAICS (Fig. 4*B*), indicating they did not differ significantly in their ability to homo-oligomerize. These results suggest once again that C-terminal tagging of PAICS-2×Strep severely impaired its heteromeric interactions, consistent with our BiFC results.

In addition to the DNPB enzymes, we also examined whether PAICS interacted with other enzymes whose activities contribute to the DNPB pathway. In particular, we wondered whether PAICS interacts with MTHFD1, which supplies the essential CHO-THF co-factor for DNPB, and whose participation in the purinosome is anticipated based on the preferential uptake of mitochondrial formate in channeled DNPB (26). We found MTHFD1 co-precipitates with PAICS in crPAICS::2×Strep-PAICS under both purine-depleted and purine-rich conditions, but as was the case with DNPB enzymes, we could not detect MTHFD1 in anti-StrepTag pull-downs from crPAICS::PAICS-2×Strep.

### Metabolic profiling reveals differences in DNPB flux between crPAICS::2×Strep-PAICS and crPAICS::PAICS-2×Strep

**Figure 5:**
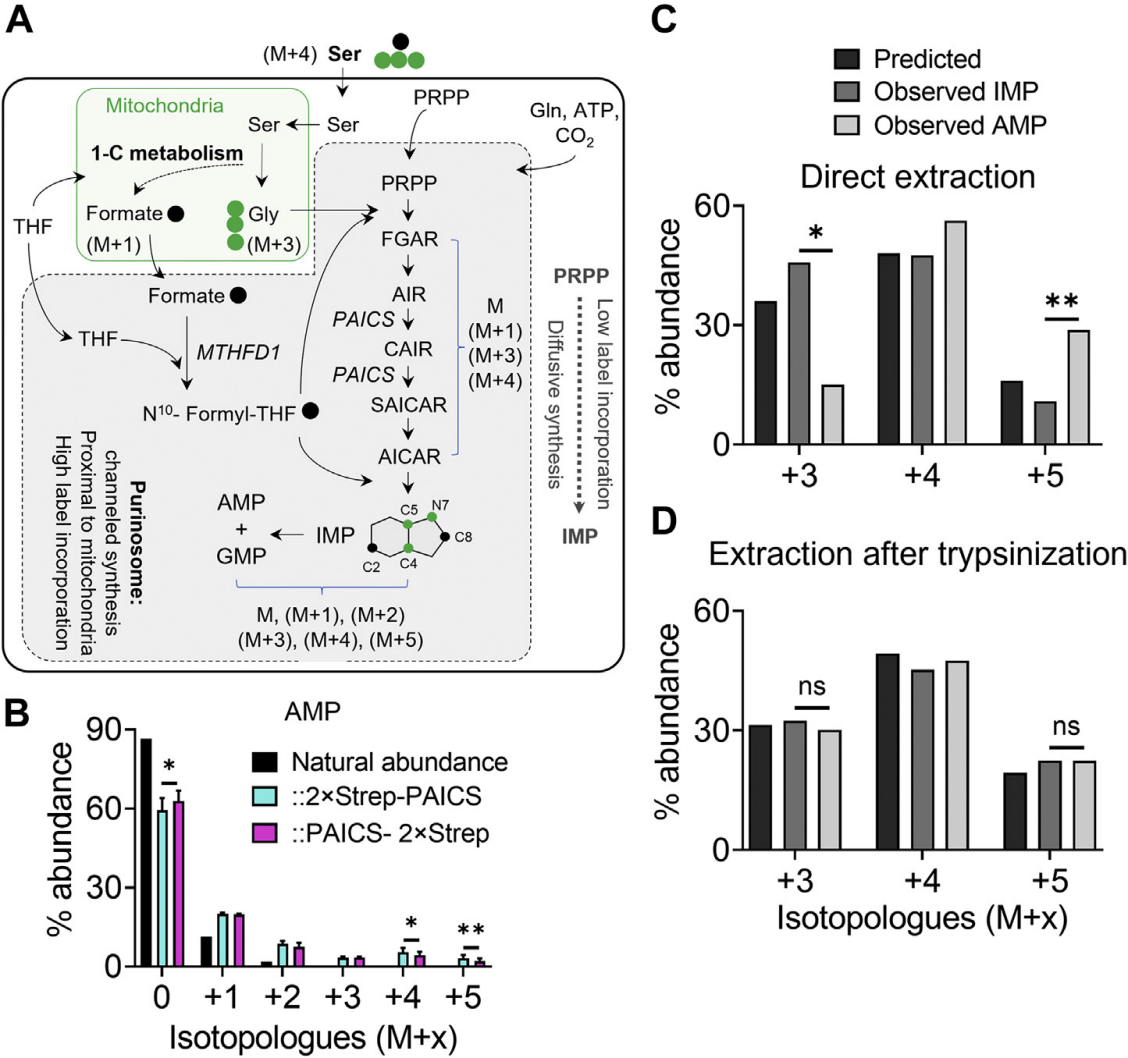
**Evidence of channeled *de novo* purine synthesis in crPAICS::2×Strep-PAICS.***A*, schematic describing incorporation of ^13^C_3_, ^15^N serine–derived ^13^C_2_, ^15^N glycine (*green circles*) and ^13^C formate (*black circles*) into the DNPB intermediates and product nucleotides. Enzyme names are italicized. *B*, comparison of the observed AMP isotopologue distribution in crPAICS::2×Strep-PAICS (*cyan bars*) and crPAICS::PAICS-2×Strep (*magenta bars*) with natural isotopologue abundance (*black bars*) confirm incorporation of ^13^C_2_, ^15^N glycine and ^13^C formate in these cell lines. Data from three independent experiments was used for paired *t* tests to compare the isotopologue distribution in crPAICS::2×Strep-PAICS *versus* crPAICS:PAICS-2×Strep. *C*, in crPAICS::2×Strep-PAICS, the isotopologue distribution of newly synthesized AMP (*light gray bars*) is significantly different from that of IMP (*dark gray bars*) and the pattern predicted based on the percentage ^13^C formate incorporation in the intermediate FGAR (*black bars*). A significantly lower fraction of the +3 isotopologue and a significantly higher fraction of +5 isotopologue, respectively, in ATP compared to IMP is a hallmark of channeled DNPB by mitochondria-associated purinosomes. *D*, purinosome integrity in crPAICS::2×Strep-PAICS was disrupted by trypsinization, releasing the purinosome-bound pools of all intermediates. After trypsinization, IMP and ATP show similar isotopologue distribution patterns. In (*C*) and (*D*), values from a representative experiment are shown, one-tailed paired *t* test was performed with values from three independent experiments; *p*-value: ‘ns’- not significant, ‘∗’ <0.05, ‘∗∗’ <0.01. DNPB, *de novo* purine biosynthesis; FGAR, phosphoribosyl-N-formylglycineamide; IMP, inosine monophosphate; PAICS, phosphoribosylaminoimidazole carboxylase/succinocarboxamide synthetase.

We wondered whether the disturbed PPIs of C-terminally tagged PAICS, as indicated by our BiFC and co-IP results, correlates with disturbed DNPB activity. The fact crPAICS::PAICS-2×Strep proliferates more slowly than crPAICS::2×Strep-PAICS, despite a high expression of PAICS protein, suggests that this is likely to be the case. We therefore conducted metabolic profiling of these two rescued cell lines to compare their DNPB activities. Rescued cells were grown in purine-depleted, glycine- and serine-free media and labeled by supplementing with ^13^C_3_ and ^15^N serine (30 μM) for 6 and 8 h, respectively. In cancer cells, owing to the compartmentalization of one-carbon metabolism, serine is metabolized by the mitochondrial serine hydroxymethyltransferase (SHMT2), bifunctional methylenetetrahydrofolate dehydrogenase/cyclohydrolase (MTHFD2/2L), and formyltetrahydrofolate synthetase (MTHFD1L) to produce glycine and formate (Fig. 1). Therefore, under our experimental conditions, mitochondria are the primary sources of ^13^C_2_, ^15^N glycine and ^13^C formate. Mitochondria-associated purinosmes, carrying out channeled purine synthesis, are expected to preferentially utilize mitochondrially derived substrates (Fig. 5*A*). Thus, channeled DNPB flux from purinosomes can be distinguished from synthesis carried out by freely diffusing DNPB enzymes in the bulk cytosol, by the difference in their respective isotopologue fraction distributions (26). Another feature of channeled DNPB is the preferential conversion of IMP to adenine nucleotides compared to guanine nucleotides (26). Therefore, changes in the native PPIs within the purinosome may also alter the partitioning of IMP between AMP and GMP.

Within 6 h of ^13^C_3_, ^15^N serine supplementation, both crPAICS::2×Strep-PAICS and crPAICS::PAICS-2×Strep showed significant label incorporation in AMP (Fig. 5*B*), though their overall AMP isotopologue distribution was different. To ascertain metabolic channeling, cell extracts were prepared either by direct extraction method (Fig. 5, *B* and *C*) or after trypsin treatment (Fig. 5*D*), which disrupts purinosomes and leads to the homogeneous mixing of the channeled and diffusively synthesized pools of DNPB intermediates and products (see experimental procedures). We compared the relative abundances of +3, +4, and +5 isotopologues of the newly synthesized AMP and IMP. In crPAICS::2×Strep-PAICS, upon direct extraction, the isotopologue composition of AMP was significantly different from that of IMP and the values predicted based on a diffusive synthesis model, indicating the presence of channeled DNPB (Fig. 5*C*). The difference between AMP and IMP isotopologue distribution is lost when metabolites were extracted following trypsinization, consistent with the idea that this treatment disrupts purinosome formation (Fig. 5*D*). Thus, in crPAICS::2×Strep-PAICS, a functional DNPB pathway exhibiting signatures of channeled synthesis similar to that found in WT HeLa has been reconstituted.

**Figure 6:**
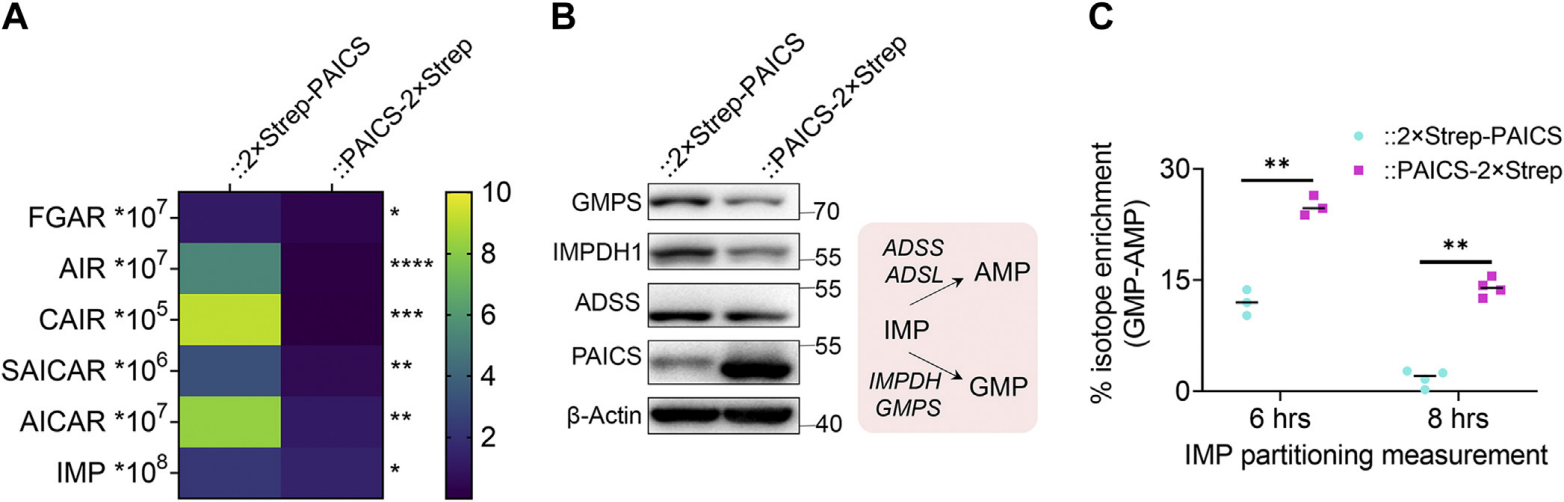
**Metabolic differences between crPAICS::2×Strep-PAICS and crPAICS::PAICS-2×Strep.** Metabolic profiling of crPAICS::2×Strep-PAICS and crPAICS::PAICS-2×Strep after trypsin treatment. *A*, crPAICS::PAICS-2×Strep shows reduced levels of all DNPB intermediates (FGAR, AIR, CAIR, SAICAR, and AICAR) and IMP when compared with crPAICS::2×Strep-PAICS. Ratio paired two-tailed *t* test was performed to compare the metabolite abundances; *p*-value: ‘∗’<0.05,‘∗∗’<0.01, ‘∗∗∗’<0.005, ‘∗∗∗∗’ <0.0005. *B*, Western blot analysis indicating crPAICS::2×Strep-PAICS and crPAICS::PAICS-2×Strep expressing similar levels of the enzymes that convert IMP to AMP (ADSS) or GMP (IMPDH, GMPS). *C*, crPAICS::PAICS-2×Strep (*magenta squares*) partitions IMP into GMP and AMP differently than crPAICS::2×Strep-PAICS (cyan circles). The difference between total isotope enrichment in GMP and AMP is significantly higher in crPAICS::PAICS-2×Strep than in crPAICS::2×Strep-PAICS, indicating that IMP is preferentially partitioned into guanine nucleotide synthesis in crPAICS::PAICS-2×Strep. The difference between the two PAICS rescue lines was consistent both upon 6 h as well as 8 h of isotope incorporation (values from three or four biological replicates are plotted, *black line* indicates median). Paired one-tailed *t* tests were performed on mean and values from three independent experiments shown above; *p*-value: ‘∗∗’ <0.01. AIR, 5-aminoimidazole ribotide; AICAR, 5-aminoimidazole-4-carboxamide ribonucleotide; CAIR, 5-phosphoribosyl-4-carboxy-5-aminoimidazole; DNPB, *de novo* purine biosynthesis; FGAR, phosphoribosyl-N-formylglycineamide; IMP, inosine monophosphate; PAICS, phosphoribosylaminoimidazole carboxylase/succinocarboxamide synthetase; SAICAR, phosphoribosylaminoimidazolesuccinocarboxamide.

However, when we tried a similar analysis for crPAICS::PAICS-2×Strep, we were stymied by the low abundance of DNPB intermediates (upon direct extraction) in this cell line. To allow the estimation of total abundance of the DNPB intermediates— phosphoribosyl-N-formylglycineamide (FGAR), 5-aminoimidazole ribotide (AIR), 5-phosphoribosyl-4-carboxy-5-aminoimidazole (CAIR), SAICAR, AICAR, and IMP—and end products, we extracted metabolites following limited trypsin treatment of cells. The total pools of DNPB intermediates and products in crPAICS::PAICS-2×Strep were found significantly lower compared to that in crPAICS::2×Strep-PAICS (Fig. 6*A*). Since purine metabolism is intricately tied to the mitochondrial one-carbon metabolism, we examined the CHO-THF– and CH_2_-THF–mediated isotope incorporation in formylmethionine and dTMP (deoxythymidine monophosphate), respectively (Fig. S3). Our analysis confirmed that the folate cofactor–mediated transfer of one carbon units for formylmethionine as well as dTMP synthesis were unaffected in the two PAICS rescue lines. The two cell lines however exhibited significant differences in their partitioning of IMP to AMP and GMP. Here, crPAICS::2×Strep-PAICS shows a greater preference toward IMP to AMP conversion, a signature of channeled DNPB (26), when compared to crPAICS::PAICS-2×Strep (Fig. 6*C*). We found that crPAICS::2×Strep-PAICS and crPAICS::PAICS-2×Strep, with the exception of PAICS, exhibit similar levels of the core DNPB enzymes (Fig. 3*C*). Western blot analysis for enzymes that convert IMP to AMP (adenylosuccinate synthase) or GMP (IMP dehydrogenase, GMP synthase) showed that crPAICS::PAICS-2×Strep, despite a greater preference for IMP to GMP conversion, actually has somewhat less expression of IMP dehydrogenase and GMP synthase (Fig. 6*B*). Thus, the metabolic disturbances found in crPAICS::PAICS-2×Strep, that extend far beyond the immediate catalytic functions of PAICS itself, are likely attributable to the C-terminal labeling of PAICS alone.

## Discussion

In this article, we have presented evidence, obtained using orthogonal methods, that the DNPB enzyme PAICS interacts with other DNPB enzymes GART, PFAS, ADSL, and ATIC, as well as with MTHFD1, which provides the essential CHO-THF cofactor for this pathway. We have shown this both in live, intact cells (BiFC) and in cells that have not been subjected to PAICS over-expression (co-IP from rescued crPAICS cells). Enzyme–enzyme interactions should be prerequisite to the formation of the purinosome; our results thus provide direct molecular evidence in support of the purinosome hypothesis. Furthermore, our results indicate the interactions of PAICS with other DNPB enzymes are present in both purine-rich and purine-depleted conditions. This raises the possibility that in the cells probed, the purinosome is assembled from subcomplexes that are present regardless of the DNPB state of the cell. It will be interesting to investigate whether this is a feature found only in rapidly proliferating cells (*e.g.*, cancer cells) or is generic to most human cell types.

It is tempting to hypothesize, based on our findings, that PAICS may have a privileged role in organizing the purinosome through its interactions with the other DNPB enzymes. However, we note that PAICS expression at a fraction of that found in WT HeLa is sufficient to support cell growth and proliferation in purine-depleted media; thus, it is likely that in many cell types, PAICS is expressed at a level in large excess of what is needed to satisfy cellular purine demand and that the fraction of PAICS molecules in complex with other DNPB enzymes maybe low. Furthermore, we cannot rule out the possibility that the heteromeric interactions observed may be indirect. If multiple DNPB enzymes, together with PAICS, are part of the same large complex, pulling down PAICS should co-precipitate all other enzymes, even if they do not directly interact with PAICS. Thus, it is crucial to determine what interactions may be revealed as we examine the remaining DNPB enzymes. Given our success with PAICS, a similar strategy to what we have employed here, applied to the remaining DNPB enzymes, should reveal further interactions. A systematic mapping of PPIs between the six DNPB enzymes should reveal important clues regarding the molecular architecture of the purinosome.

The assays we described here probes only binary PPIs; however, the success of our BiFC approach suggests a way forward toward probing higher order interactions within the purinosome. Recently, several bimolecular complementation approaches, based on the splitting/complementation of proximity labeling enzymes, such as APEX2, BioID, and TurboID, have been reported (49, 50, 51, 52). Using such a system, a functional labeling enzyme is reconstituted only when PAICS and PFAS (for example) are interacting; it is therefore well-poised to probe higher order PPIs in the purinosome. Such experiments will allow us to better distinguish the formation of binary complexes between DNPB enzymes, from the assembly of the complete purinosome. Understanding the regulation of these possibly distinct processes may prove important for elucidating how cells regulate DNPB in response to varying purine demand.

We found that the tagging of PAICS at its C terminus impaired its biological function. Our PPI assays indicate C-terminal tagged PAICS has impaired heteromeric interactions, while our metabolic experiments indicate that crPAICS::PAICS-2×Strep, when compared to crPAICS::2×Strep-PAICS, has a markedly altered metabolic profile. In crPAICS::2×Strep-PAICS, we observed metabolic signatures of channeled DNPB similar to that found in WT HeLa. By contrast, in crPAICS::PAICS-2×Strep, the overall DNPB flux is reduced, despite a six-fold higher PAICS protein expression, and the preferential partitioning of the IMP toward AMP is also reduced. The fact C-terminally tagged PAICS can stably rescue crPAICS cells indicates its essential enzymatic activities are intact. While it is still possible tagging PAICS at its C-terminus may negatively affected its enzymatic kinetics and/or ligand binding affinity, we note that based on available crystal structures (53, 54), the C-terminus of PAICS does not lie near to known ligand-binding or active sites. The crystal structure of the PAICS octamer does offer a clue as to why C-terminal tagging of PAICS might affect its PPIs. The PAICS octamer takes an approximately plate-like structure; whereas the N-terminus of PAICS appears to be highly flexible and extends from the edges of the plate, its C-terminus ends on a stable alpha helix that lies on the plate face (Fig. 3*A*). If the plate face of the PAICS octamer serves as a PPI interface, even a small C-terminal tag may provide sufficient steric hindrance to weaken binding.

The generation of stably rescued polyclonal cell lines crPAICS::2×Strep-PAICS and crPAICS::PAICS-2×Strep depended on the random integration of the transfected PAICS expression vectors (driven in both cases by the CMV promoter) into the crPAICS genome. Although the expression level of reintegrated PAICS will depend sensitively on the site of integration, long-term culture in purine-depleted media favors clones whose PAICS activity can support continued cell proliferation under that condition. The fact that crPAICS::PAICS-2×Strep expresses PAICS at a 6-fold higher level than crPAICS::2×Strep-PAICS, while the expression of the remaining DNPB enzymes are similar across these two cell lines (Figs. 3*C* and 6*B*), indicates the altered metabolic profile of crPAICS::PAICS-2×Strep, and its reduced rate of growth is attributable to C-terminal–tagged PAICS alone and that the defect in C-terminally tagged PAICS (whatever is its nature) can be partially compensated by elevated enzyme expression.

For a functional but kinetically impaired enzyme, higher concentration can compensate for kinetic defects, provided the defective enzyme has become rate limiting. In our metabolic measurements, we found no evidence that C-terminally tagged PAICS in crPAICS::PAICS-2×Strep has become limiting. For example, we did not observe an excess accumulation of DNPB intermediates upstream of PAICS in crPAICS::PAICS-2×Strep as compared to crPAICS::2×Strep-PAICS. On the other hand, for pathways (such as DNPB) where enzymes receive their substrates in a channeled manner from an interacting partner, where only a (possibly very small) fraction of enzymes are actually in complex and contribute meaningfully to the overall flux, overexpressing an enzyme whose kinetics is normal, but whose heteromeric interactions are destabilized, helps to maintain complexation by increasing the *k*_on_ of the complex as compensation for the reduced complex stability (increased *k*_off_).

The principal rationale of the purinosome hypothesis is that the complexation is essential for optimal DNPB flux. This idea is difficult to explore directly because conditions that would disrupt the purinosome, while retaining the activities of individual DNPB enzymes, are unknown. We believe the comparison of the two stably rescued crPAICS cell lines provides a natural test of this central aspect of the purinosome hypothesis. Our data give no indication that the enzymatic activities of C-terminal–labeled PAICS are compromised, while providing evidence that its heteromeric interactions with other DNPB enzymes have been weakened and that this is correlated with reduced DNPB intermediate pools and a perturbed partitioning of IMP toward AMP and GMP. Taken together, our results not only provide direct molecular evidence, in the form of PPIs, in support of the purinosome hypothesis, we believe they also indicate that the disruption of these interactions has a profoundly negative impact on the output of this important metabolic pathway.

## Experimental procedures

### BiFC assay and signal quantification

#### BiFC assay of enzyme pairs

10^5^ HeLa cells were plated on glass-bottomed 35 mm TC dishes (Cellvis) in 1.5 ml FluoroBrite Dulbecco's modified Eagle's medium (DMEM) (ThermoFisher) supplemented with l-glutamine, sodium pyruvate, and normal (purine-rich) or dialyzed (10 kDa MWCO) FBS (purine-depleted) (Atlanta Biologics). After 48 h, the media was replaced with 1 ml fresh media, and the cells are transfected (Xfect DNA Transfection Reagent, TakaraBio) with two plasmids (2.25 μg each) expressing complementarily tagged (SYFP2_1-215_ and SYFP2_210-238_) enzymes (whose interactions we wish to assess), along with a plasmid (0.5 μg) expressing LSSmOrange (serving as a transfection marker). Twenty-four hours posttransfection, cells are imaged (at 60X) under an epifluorescence microscope (Nikon TE Eclipse 2000) equipped with a stage-top incubation system (Tokai HIT) that maintains the cells at 37 °C in a 5% CO2 atmosphere. Cells are imaged using a 60/1.2 NA oil immersion objective in both LSSmOrange and YFP channels. A preliminary assay identified combinations of enzyme pairs/labeling termini pairings that gave clear BiFC signals; these are then subjected to detailed quantitative analysis.

#### BiFC image analysis

For each sample, 15 to 20 images, each with 5 to 10 LSSmOrange-positive cells were acquired. For each image, we measure the mean YFP (SYFP2 BiFC) fluorescence intensity of all pixels whose LSSmOrange signal is above threshold (set by imaging untransfected HeLa cells). Pixels whose LSSmOrange signal is below threshold are designated as background pixels. The BiFC score is calculated as

$$\text{BiFC}\;\text{signal}\;=\frac{\left\langle \text{YFP}|\text{LSSmOrange}>\text{threshold}\right\rangle }{\left\langle \text{YFP}|\text{LSSmOrange}\leq \text{threshold}\right\rangle }\;-1\;$$

Images analyses were conducted using custom-written macros in Fiji/ImageJ. Analyses were repeated in triplicate. For the P2A BiFC controls, we found all control pairs gave essentially the same background. The baseline shown in Figure 2*D* represents the average ± standard deviation of all the P2A controls, repeated in triplicate.

### Generation and characterization of stably rescued crPAICS cell lines

#### Generation of stably rescued crPAICS cell lines

crPAICS cells are plated in 6-well TC plate at a density of 2 × 10^5^ cell/well and cultured in DMEM, supplemented with 10% fetal bovine serum and 30 μM adenine, for 48 h. The cells are switched to purine-depleted media and immediately transfected with 5 ug of plasmid vector (pC1-Neo PAICS-2×Strep or pC1-Neo 2×Strep-PAICS) using Xfect DNA Transfection Reagent (TakaraBio). Hereafter, the culture media is replaced with fresh purine-depleted (DMEM, supplemented with 10% dialyzed fetal bovine serum, 10 kDa MWCO) media every 2 days. After 7 to 10 days, the transfected cells are trypsinized and passaged into T25 flasks and maintained in purine-depleted media (replaced every 2–3 days) for 4 to 6 weeks. During this period, most of the crPAICS cells die and lift off; however, colonies of proliferating cells also emerge, indicating the transfected PAICS expression vector has been reintegrated into the genome. Once the proliferating cell colonies have taken over the cell culture, they are deemed to be stably rescued and designated as P1. Hereafter, the rescued cells are maintained in purine-depleted media to avoid the loss of reintegrated PAICS expression (*e.g.*, due to promoter silencing).

#### Cell-growth assay

On day 0, stably rescued crPAICS::2×Strep-PAICS and crPAICS::PAICS-2×Strep of similar passage (between 5 and 10) are seeded in 6-well TC plates at a density of 5 × 10^4^ cells/well in purine-depleted media. The media is replaced with fresh media on days 2, 4, 6, 7, 8, and 9, and one set of cells is collected every 2 days. To collect the sample, cells are fixed in 0.2% glutaraldehyde (Electron Microscopy Sciences) in PEM buffer (100 mM Pipes, 1 mM EGTA, 1 mM MgCl_2_, pH 6.8) for 15 min at room temperature. The fixed cells are washed in three changes of tris-buffered saline (TBS) for a total of 30 min. Following the washes, the fixed cells are incubated in DAPI/TBS (5 μg/ml) for 30 min and then imaged using epifluorescence microscopy at low magnification (5×). For each sample, we acquired 25 fields (3.2 mm × 3.2 mm) of view. The images are then processed in Fiji/ImageJ to identify and count DAPI-stained cell nuclei using a custom-written code. Experiments are conducted in triplicate.

#### Immunofluorescence analysis of PAICS expression in single cells

WT HeLa, crPAICS, crPAICS::2×Strep-PAICS, and crPAICS::PAICS-2×Strep cells are seeded onto glass-bottomed 35 mm TC dishes at a density of 10^5^ cells/dish. After 48 to 72 h, cells are fixed in 4% paraformaldehyde (Electron Microscopy Sciences) in PEM buffer for 5 min at room temperature, followed by further fixation in 4% paraformaldehyde in borate buffer (100 mM sodium borate, 1 mM EGTA, 2 mM MgCl_2_, pH 11) for 10 min at room temperature. Fixed cells are washed (3 washes, 5 min/wash) with PBS and TBS and permeabilized with 0.5% SDS in TBS (10 min at room temperature); SDS treatment is also necessary to partially denatured cellular proteins, thereby exposing targeted epitopes to the anti-PAICS antibody used. Permeabilized cells are washed with TBS (3 washes), blocked (1 h at room temperature) in IF buffer (5% normal donkey serum, 1% BSA, in TBS), then incubated (1 h at room temperature) with anti-PAICS primary antibody (Bethyl A304-547A, 1:2000 in IF buffer). Afterward, cells are washed with high-salt (600 mM NaCl) TBS (3 washes, 10 min/wash) and normal TBS (3 washes, 5 min/wash), before being incubated (30 min at room temperature) with fluorescently labeled secondary antibodies (CF488A-conjugated donkey anti-rabbit antibody, Biotium 20015, 1:1000 in IF buffer). Stained cells are washed with high-salt TBS and normal TBS (3 washes each), counterstained with DAPI (5 μg/ml in TBS, incubated 30 min at room temperature), and imaged in Tris buffer (50 mM Tris, pH 7.8) using epifluorescence microscopy. For each sample, 30 to 50 images, containing several hundred cells, were collected. Images were analyzed using Fiji/ImageJ to measure immunofluorescence intensities of individual cells.

### Co-immunoprecipitation and Western blot analysis

#### Anti-StrepTag affinity purification

Cells are trypsinized, washed, and pelletized. The cell pellet is lysed in lysis/coIP buffer containing Tris (50 mM, pH 7.8), KCl (135 mM), NaCl (15 mM), MgCl_2_ (5 mM), Triton X-100 (1% v/v), and supplemented with DTT (1 mM), ATP (2 mM), l-glutamine (1 mM), protease (Roche cOmplete Protease Inhibitor Cocktail without EDTA), and phosphatase (Pierce HALT Phosphatase Inhibitor Cocktail) inhibitors. The cell lysate is clarified by centrifugation (10 min at 10,000*g*, 4 °C), and the total protein concentration is determined by Bradford assay. Clarified lysate is affinity-purified (30 min at 4 °C) using MagStrep “type 3” XT anti-StrepTag beads (IBA Lifesciences) at a concentration of 25 μl beads per 4 × 100 mm dishes. The beads are then washed with lysis/co-IP buffer and eluted by boiling (5 min) in 30 μl Laemmli buffer.

#### SDS-PAGE gel electrophoresis and Western blot analysis

For SDS-PAGE gel electrophoresis, equal volumes of elution product per lane are loaded onto polyacrylamide gels for electrophoresis. Afterward, the proteins are transferred to the polyvinylidene difluoride membrane. The membrane is blocked in 5% dry milk in TBS for 1 h at room temperature, then probed with primary (incubated overnight at 4 °C) and HRP-conjugated secondary antibodies (incubated 1 h at room temperature). The membranes are imaged on BioRad Gel Doc imager using SuperSignal West Pico Plus chemiluminescent substrate (Thermo Scientific). A list of antibodies and dilutions used is given in Table S3.

### ^13^C_3_, ^15^N serine incorporation, metabolite extraction, and LC/MS analysis

To estimate the total purine flux and the channeled DNPB flux by mitochondria-associated purinosmes, ^13^C_3_, ^15^N serine (Cambridge isotope laboratory) incorporation into DNPB intermediates and nucleotide products and metabolite extraction was performed as described in (26). Cells were maintained in purine-depleted DMEM media (with 10% dFBS). Three to four million cells were seeded/10 cm culture dish and allowed to grow for 24 h in RPMI (with 10% dFBS), after which, the media was changed to purine-depleted MEM (without serine and glycine, with 10% dFBS). The media was supplemented with ^13^C_3_, ^15^N serine to a final concentration of 30 μM, and isotope incorporation was allowed for 4 or 8 h, following which cells were gently washed thrice with 1× PBS. Cells were harvested either by scraping after directly adding cold 80% methanol (−20 °C) onto the culture dish or by trypsinization followed by PBS wash and the addition of 80% methanol. Direct addition of 80% methanol is known to cause rapid quenching of all enzymatic reactions and restrict release of the intermediates from within the purinosome complex, while trypsinization leads to disassembly of the purinosome complex and release of all the bound intermediates in the cytosolic bulk. Metabolite extraction was performed as described in (26) and extracts were dried under N_2_ gas flow. The dried extracts were reconstituted in 3% methanol with 1 μM chlorpropamide (internal standard) such that the final solution contained extract from ∼1 million cells/10 μl. 10 to 15 μl of each extract were run on a Phenomenex Hydro-RP C18 column (100 × 2.1 mm, 3 m particle size) using a water/methanol gradient (tributylamine and acetic acid added to the aqueous mobile phase and analyzed by Exactive plus orbitrap mass spectrometer controlled by Xcalibur 2.2 software (all from Thermo Fisher Scientific). Details of the gradient and run method can be found in (26) and the respective m/z values and retention time for all the analytes are given in Table S1. The expected difference in the atomic mass unit (*Δ amu*) upon incorporation of ^13^C_3_, ^15^N serine-derived ^13^C_2_, ^15^N glycine and ^13^C formate incorporation into different analytes is given in Table S2. In each experiment, three or four independent experiments were performed, and the peak area for all the analytes was used for statistical analysis.

In a pilot experiment, we confirmed that the PAICS CRISPR/Cas9 HeLa cell knock-out used for generating the rescue cell lines had no residual PAICS activity (Fig. S2*A*). While the intermediates upstream of PAICS (FGAR, FGAM, and AIR) were observed, crPAICS cells showed no detectable SAICAR or AICAR. The downstream purine nucleotides (AMP) showed no isotope incorporation. In the cell lines generated by genomic integration of the N-term PAICS, all the intermediates and products downstream of PAICS (SAICAR, AICAR, IMP, and AMP) showed significant isotope incorporation (Fig. S2*B*). This confirmed that the reintegrated PAICS construct produced catalytically active PAICS.

We also estimated the level of folate cofactor–mediated isotope incorporation in other pathways that rely on the mitochondrial one-carbon metabolism (Fig. S3). For this, we examined the ^13^C incorporation in formylmethionine and dTMP, both at the 6 and 8 h time points (Fig. S3, *B* and *C*). While formylmethionine utilizes CHO-THF within mitochondria, dTMP synthesis relies on the availability of CH_2_-THF in cytosol.

#### Data analysis

Data were plotted, and statistical tests performed using GraphPad Prism 9. To find if the PAICS rescue cell lines exhibit metabolic signature of channeled DNPB by mitochondria-associated purinosomes, within each sample, the isotopologue abundance distribution of the newly synthesized end-product nucleotides (IMP, ATP, GTP) and that expected assuming a diffusive model for DNPB (26) were compared. The diffusive model uses the respective peak areas of different FGAR isotopologues to predict the isotopologue distribution in the newly synthesized IMP and downstream nucleotide products. The model predicted isotopologue distribution of the +3, +4, and +5 species was compared with the observed distribution in IMP and ATP. Total abundance of each analyte refers to the combined peak areas for all the isotopologue species.

For calculating the difference in percentage isotope incorporation in GMP and AMP, the newly synthesized GMP and AMP were calculated as described in (26). Since the overall GMP pools are 8- to 10-fold smaller than AMP, the difference in isotope incorporation percentage (GMP-AMP) is always a positive number. Also, as isotope incorporation is performed for longer time, due to increase in equilibration of cytosolic pools of various labeled substrates, the differences between the channeled and diffusively generated pools start to merge and thus also the percentage isotope incorporation (GMP-AMP) (Fig. 6*C*).

## Data availability

All the relevant data and plasmids used in this study are available upon request.

## Supporting information

This article contains supporting information

## Conflict of interest

The authors declare that they have no conflicts of interest with the contents of this article.
