# Supplementary material for: Multienzyme interactions of the de novo purine biosynthetic protein PAICS facilitate purinosome formation and metabolic channeling
Source: J Biol Chem. 2022 Mar 21;298(5):101853. doi: 10.1016/j.jbc.2022.101853 (PMC9035706; doi:10.1016/j.jbc.2022.101853)
Supplement: Supplemental Figures S1–S3 and Tables S1–S2 [file mmc1.docx]

#### **SUPPORTING INFORMATION**

**Multienzyme interactions of the *de novo* purine biosynthetic protein PAICS facilitate purinosome formation and metabolic channeling**

Jingxuan He^1, ‡^, Ling-Nan Zou^2, ‡^, Vidhi Pareek^2^, and Stephen J. Benkovic^1*^

^1^Department of Chemistry, The Pennsylvania State University, University Park, PA 16802

^2^Huck Institutes of the Life Sciences, The Pennsylvania State University, University Park, PA 16802

**Supplementary Figure S1.** IF images showing anti-PAICS staining in wild type HeLa, crPAICS, and the rescued cell lines crPAICS::2×Strep-PAICS and crPAICS::PAICS-2×Strep. The heterogeneity of PAICS expression in the rescued cell lines presumably arises from their polyclonality. Scale bar = 25 µm.

**
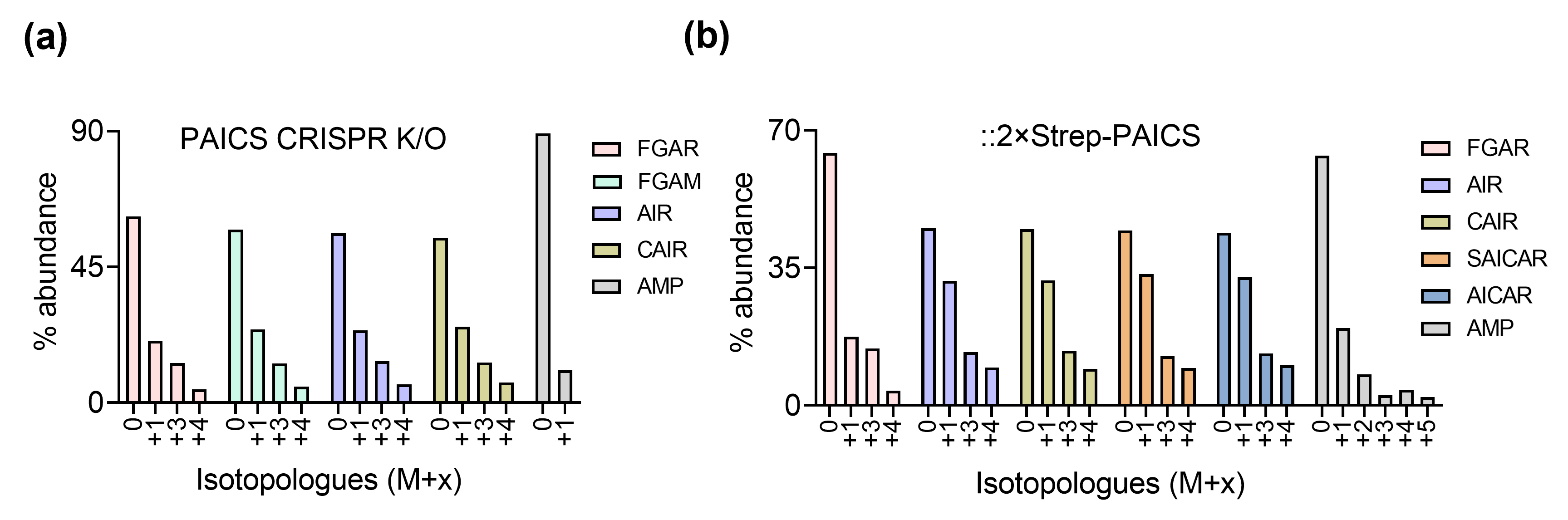
**

**Supplementary Figure S2. (a)** HeLa PAICS CRISPR/ Cas9 knock-out (K/O) cells showed label incorporation in the intermediates (FGAM, FGAM, and AIR) upstream of PAICS activity. No detectable SAICAR and AICAR were observed and no isotope incorporation was observed in AMP after six hrs incubation with ^13^C_3_, ^15^N Ser. The small amount of CAIR observed arises as a result of spontaneous carboxylation of AIR. **(b)** The polyclonal HeLa cell population generated by genomic integration of N-terminal tagged PAICS show successful incorporation of labeled ^13^C_2_, ^15^N glycine and ^13^C formate incorporation in PAICS product SAICAR and the downstream product AICAR. Acronyms- FGAR: phosphoribosyl-N-formylglycineamide, FGAM: formylglycinamidine ribonucleotide, AIR: 5-aminoimidazole ribonucleotide, CAIR: carboxyaminoimidazole ribonucleotide, SAICAR: phosphoribosyl aminoimidazole succinocarboxamide, AICAR: 5-aminoimidazole-4-carboxamide ribonucleotide, AMP: adenosine monophosphate.

**
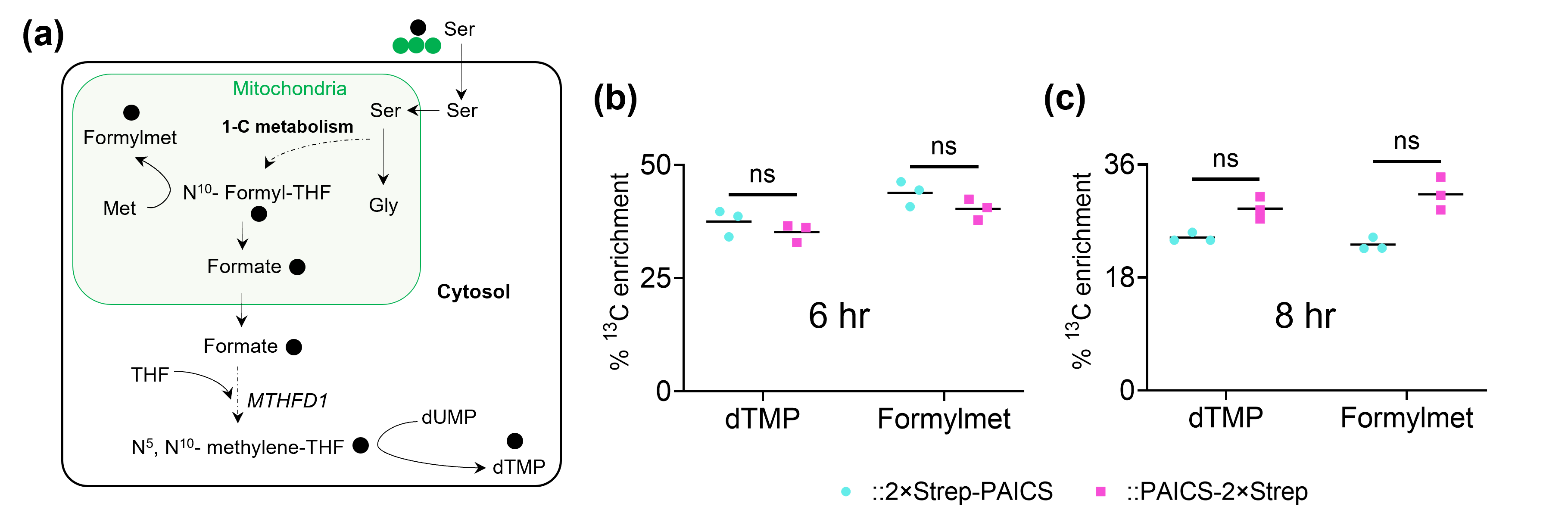
**

**Supplementary Figure S3. (a)** Schematic showing utilization of serine to produce labeled tetrahydrofolate (THF) cofactors N^10^- formyl THF and N^5^, N^10^- methylene THF in a mitochondrial one carbon (1-C) metabolism dependent manner. The formate generated inside mitochondria is transported to cytosol and is converted to N^10^- formyl THF by the cytosolic methylenetetrahydrofolate dehydrogenase/cyclohydrolase (MTHFD1). Two metabolites, deoxythymidine monophosphate (dTMP) or formylmethionine (formylmet), that utilize the mitochondrially generated one-carbon units were examined. dUMP: deoxyuridine monophosphate, methionine: Met, glycine: Gly, serine: Ser. ^13^C enrichment in dTMP and formylmethionine after **(b)** 6 hr or **(c)** 8 hr incubation of N-terminal- tagged PAICS (N-term, blue circles) and C-terminal- tagged PAICS (C-term, red squares) with labeled ^13^C_3_, ^15^N Ser. Two tailed t-test were performed using values from three independent experiments as shown in the figure; ‘ns’- not significant corresponding to p-value >0.05. No difference in the ^13^C isotope enrichment was observed for these metabolites.

**Supplementary Table 1. Metabolites with their observed retention time (min), the calculated m/z for [M-H]^-1^, and the ionic molecular formula.**

| **Metabolite** | **Elution time (min)** | **Calculated mass [M-H]^-1^** | **Molecular formula** |
| --- | --- | --- | --- |
| ATP | 15.12 | 505.9885 | C10H15N5O13P3 |
| AMP | 11.63 | 346.0558 | C10H13N5O7P |
| GTP | 15.0 | 521.9834 | C10H15N5O14P3 |
| GMP | 10.5 | 362.0507 | C10H14N5O8P |
| IMP | 10.35 | 347.0398 | C10H12N4O8P |
| SAICAR | 14.45 | 453.0664 | C13H18N4O12P |
| AICAR | 10.3 | 337.0555 | C9H14N4O8P |
| Chlorpropamide | 16.8 | 275.0267 | C10H12ClN2O3S |
| dTMP | 11.4 | 321.0493 | C10H15N2O5P |
| Formylmethionine | 12.5 | 176.0386 | C6H10NO3S |
| FGAR | 7.2 | 313.045 | C8H15N2O9P |
| CAIR | 12.7 | 338.04 | C9H13N3O9P |
| AIR | 1.9 | 294.05 | C8H13N3O7P |
| FGAM | 1.7 | 312.06 | C8H15N3O8P |

**Supplementary Table 2. The atomic mass unit difference (𝞓 *amu* ) upon incorporation of the respective isotope labeled atoms or molecule.** The values were used to compute the exact molecular weight of the labeled intermediates and end products of the DNPB pathway.

| **Molecule/atom** | **Δ amu** |
| --- | --- |
| ^13^C_2_, ^15^NGly | 3.0037 |
| ^13^C | 1..0033 |
| ^15^N | 0.997 |

**Supplementary table 3. List of antibodies used in this work**

| **primary antibodies** | **species** | **dilution** | **suppliers** |
| --- | --- | --- | --- |
| PAICS, A304-547A | rabbit | 1:2500 | Bethyl Laboratories |
| PFAS, A304-220A | rabbit | 1:2500 | Bethyl Laboratories |
| GART, A304-311A | rabbit | 1:2000 | Bethyl Laboratories |
| ATIC, A304-271A | rabbit | 1:2000 | Bethyl Laboratories |
| PPAT, LS-B9407 | mouse | 1:2500 | LSBio |
| ADSL, A304-778A | rabbit | 1:2000 | Bethyl Laboratories |
| PFAS, 76957 | rabbit | 1:1000 | Cell Signaling |
| MTHFD1, A305-286A | rabbit | 1:2000 | Bethyl Laboratories |
| GMPS, A302-417A | rabbit | 1:2000 | Bethyl Laboratories |
| ADSS, 16373-1-AP | rabbit | 1:1000 | Proteintech |
| IMPDH1, ab33039 | rabbit | 1:1000 | Abcam |
| Strep-tag, 71590 | mouse | 1:2500 | Millipore Sigma |
| β-Actin, 10R-2927 | mouse | 1:2500 | Fitzgerald |
| **secondary antibodies** | **species** | **dilution** | **suppliers** |
| Anti-rabbit HRP, 7074 | goat | 1:2000 | Cell Signaling |
| Anti-mouse HRP, 7076 | horse | 1:2000 | Cell Signaling |
